# Metformin Induces Different Responses in Clear Cell Renal Cell Carcinoma Caki Cell Lines

**DOI:** 10.3390/biom9030113

**Published:** 2019-03-22

**Authors:** Mazhar Pasha, Siveen K. Sivaraman, Ronald Frantz, Abdelali Agouni, Shankar Munusamy

**Affiliations:** 1 Pharmaceutical Sciences Department, College of Pharmacy, Qatar University, P.O. Box 2713, Doha, Qatar; pashamazhar@gmail.com; 2 Interim Translational Research Institute, Academic Health System, Hamad Medical Corporation, P.O. Box 3050, Doha, Qatar; SSivaraman@hamad.qa; 3 Department of Pharmaceutical and Administrative Sciences, College of Pharmacy and Health Sciences, Drake University, Des Moines, IA 50311, USA; ronald.frantz@drake.edu

**Keywords:** renal cell carcinoma, von Hippel-Lindau, hypoxia-inducible factor, AMP-activated kinase, metformin

## Abstract

Clear cell renal cell carcinoma (ccRCC) is the most common and lethal form of urological cancer diagnosed globally. Mutations of the von Hippel-Lindau *(VHL)* tumor-suppressor gene and the resultant overexpression of hypoxia-inducible factor (HIF)-1α protein are considered hallmarks of ccRCC. Persistently activated HIF-1α is associated with increased cell proliferation, angiogenesis, and epithelial–mesenchymal transition (EMT), consequently leading to ccRCC progression and metastasis to other organs. However, the *VHL* status alone cannot predict the differential sensitivity of ccRCC to cancer treatments, which suggests that other molecular differences may contribute to the differential response of ccRCC cells to drug therapies. In this study, we investigated the response to metformin (an antidiabetic drug) of two human ccRCC cell lines Caki-1 and Caki-2, which express wild-type *VHL*. Our findings demonstrate a differential response between the two ccRCC cell lines studied, with Caki-2 cells being more sensitive to metformin compared to Caki-1 cells, which could be linked to the differential expression of HIF-1α despite both cell lines carrying a wild-type *VHL*. Our study unveils the therapeutic potential of metformin to inhibit the progression of ccRCC in vitro. Additional preclinical and clinical studies are required to ascertain the therapeutic efficacy of metformin against ccRCC.

## 1. Introduction

Renal cell carcinoma (RCC) is the third most common urological cancer and the sixth leading cause of cancer deaths in the United States [1,2,3,4]. The renal cancer cells are extremely proliferative and metastatic, and account for 3.8% of all new cancers [1,3]. Clear cell renal cell carcinoma (ccRCC) is the most common subtype of RCC and accounts for approximately 70–75% of all cases [5]. For localized ccRCC, surgical resection continues to be a curative treatment option [6,7,8]. However, for the management of recurrent or metastatic ccRCC, targeted drug therapy is preferred; this includes the use of tyrosine kinase inhibitors such as sunitinib, sorafenib, axitinib and pazopanib; mammalian target of rapamycin (mTOR) inhibitors such as temsirolimus and everolimus; and monoclonal antibodies such as bevacizumab, which targets vascular endothelial growth factor (VEGF) [9,10]. In addition to tyrosine kinase inhibitors, immunotherapy using nivolumab and interferon-α are considered to be the standard treatment strategies [11,12]. However, ccRCC gradually develops resistance to chemotherapy and other treatment modalities such as hormonal therapy and radiotherapy; hence, there is a tremendous need for the advancement of new therapeutic alternatives [13]. 

Mutation of the von Hippel-Lindau (*VHL*) tumor suppressor gene and the resultant overexpression of hypoxia-inducible factor (HIF)-1α protein are considered hallmarks of ccRCC [8,14]. Persistently activated HIF-1α is associated with cell proliferation, angiogenesis, and epithelial–mesenchymal transition (EMT), consequently leading to ccRCC progression and metastasis to other organs [6,8]. Several cell lines have been used to model ccRCC in research with some expressing wild-type *VHL,* and others characterized by a loss-of-function mutation in *VHL* [15]. The *VHL* status has been reported in several studies to affect the sensitivity of ccRCC cells to various drug therapies; however, multiple lines of evidence suggest that other molecular differences may also contribute to the differential sensitivity of RCC cells to drugs [16,17,18]. In this study, we focused on investigating some of the molecular differences between two major cell lines used in ccRCC, namely Caki-1 and Caki-2. Both Caki-1 and Caki-2 cells are primarily defined as human ccRCC cell lines; however, Caki-1 cell lines are metastatic ccRCC, harboring wild-type *VHL*, whereas, Caki-2 cell lines are considered as ccRCC expressing wild-type von Hippel-Lindau protein (pVHL) [15].

Metformin, an activator of the AMP-activated kinase (AMPK), is primarily prescribed for the management of type-2 diabetes. Specifically, metformin acts through inhibiting gluconeogenesis (glucose production) in the liver via activation of AMPK signaling in hepatocytes [19]. In addition, metformin also inhibits pyruvate dehydrogenase activity and mitochondrial respiration, which in turn increases the theoretical risk of lactic acidosis in cells [19]. Nevertheless, the incidence of lactic acidosis is rare and is observed mainly in patients with severe renal dysfunction [19,20]. Thus, a lower dose of metformin is recommended in diabetic patients with moderate kidney disease, and the use of metformin is contraindicated in patients with advanced kidney disease [19,20]. Emerging studies suggest that metformin exerts antineoplastic effects in various types of cancers, such as cancers of the breast, colon, liver, prostate, and kidney [21,22,23]. Although the activation of AMPK by metformin has been shown to decrease the expression of HIF-1α and mTOR activation in some cancers and in diabetic rat kidneys [23,24,25], comprehensive signaling pathways exerted by metformin to inhibit cell proliferation, cell cycle arrest and apoptosis in ccRCC are yet to be thoroughly investigated.

Work by Xu et al. [26] found that the downregulation of HIF-1α by small hairpin RNA (shRNA) inhibited cell growth, migration, and invasion in Caki-2 and OS-RC-2 cells, and suppressed tumorigenicity in a xenograft mouse model. Similarly, Kondo et al. [27] reported that the downregulation of HIF-2α using shRNA in 786-O and A498 cells inhibited their tumorigenicity in a xenograft mouse model. 

Intriguingly, Raval et al. [17] demonstrated contrasting properties of HIF-1α and HIF-2α in *VHL*-defective ccRCC cells, with HIF-1α inhibiting and HIF-2α promoting cell growth. Conversely, Gudas et al. [28] reviewed the evidence available from human ccRCC cell lines, human ccRCC specimens, murine models, and mouse xenograft models, and reported a convergence of both clinical and theoretical data highlighting the significant role of HIF-1α in promoting ccRCC. Furthermore, the authors noted that ccRCC is a highly heterogeneous disease, the exact roles of HIF-1α and HIF-2α are incomplete, and further research needs to focus on determining their diverse role in ccRCC. Altogether, these previous studies highlight the complexity of ccRCC and the need for further dissection of the signaling pathways involved in ccRCC tumorigenesis and survival. The *VHL* gene is often mutated in ccRCC cell lines (e.g., 786-O and UM-RC-2) with subsequent activation of the HIF pathway that regulates the expression of various target proteins involved in ccRCC progression; however, the status of *VHL* alone cannot predict the differential sensitivity of ccRCC to cancer treatments. Therefore, it is believed that other molecular differences may contribute to the differential response of these cells to drug therapies. Thus, it is of paramount importance to decipher the critical molecular pathways contributing to ccRCC progression.

Liu et al. [3] observed that metformin effectively induced G0/G1 cell phase arrest and suppressed cell growth in 786-O and OS-RC-2 cell lines, and an in vivo murine model of RCC. Similarly, Kalogirou et al. [29] revealed that Caki-1 cells were less sensitive towards metformin treatment in comparison to Caki-2 cells, and that the sensitivity of metformin was associated with microRNA-21 (miR-21)/phosphatase and tensin homolog (PTEN) tumor suppressor expression in both Caki-1 and Caki-2 cells. Although accumulating evidence suggests that metformin inhibits cell proliferation in some cancers, the precise mechanism(s) exerted by metformin to inhibit the growth of ccRCC remain(s) unclear and yet to be fully elucidated. Therefore, the aim of this work was to investigate the antineoplastic effect of metformin against ccRCC cell lines, namely Caki-1 and Caki-2, and to explore if there is a differential selectivity in the *VHL* status of these two cell lines by evaluating HIF-1α and HIF-2α expression. In addition, we aimed to explore other critical downstream targets and their possible underlying signaling mechanisms contributing to the progression of ccRCC such as phosphoinositide 3-kinase (PI3K)/AKT/mTOR, autophagy, and Wnt/β-catenin pathways, and assess any possible differential activation of these signaling hubs between Caki-1 and Caki-2 cells.

## 2. Materials and Methods 

### 2.1. Reagents

Metformin (1,1-dimethylbiguanide hydrochloride) was purchased from Sigma-Aldrich (St. Louis, MO, USA) and phosphate-buffered saline (PBS) (Gibco, Grand Island, NY, USA) was used to solubilize it. The various concentrations of metformin used were 1, 2, 5, 10, 20, and 50 mM diluted in culture media. McCoy’s 5A (modified) medium, fetal bovine serum (FBS), 0.25% Trypsin– ethylenediaminetetraacetic acid (EDTA) solution, penicillin/streptomycin (10,000 U/mL) were purchased from Gibco. Alamar Blue™ cell viability reagent and Tali™ cell cycle kit were purchased from Thermo-Fisher Scientific (Eugene, OR, USA). Antibodies used for Western blot analysis were procured from the following sources: HIF-1α, phospho-AMPK (Thr172), phospho-mTOR (Ser2448), phospho-Akt (Ser473), α-SMA, LC3-II, phospho-PTEN(Ser380), phospho-GSK-3β (Ser9), Wnt3a, phospho-LRP6 (Ser1490), phospho-β-Catenin (Ser33/37/Thr41), and horseradish peroxidase-conjugated secondary antibodies were purchased from Cell Signaling Technology (Danvers, MA, USA), and β-actin antibody was from Abcam (Cambridge, MA, USA). For flow cytometry analysis, fluorescein isothiocyanate (FITC)-labeled annexin V and propidium iodide (PI) staining solution were purchased from BD Biosciences (San Jose, CA, USA) and Cyto-ID^®^ autophagy detection kit from Enzo Life Sciences, Inc. (Farmingdale, NY, USA). All other reagents were purchased from Sigma-Aldrich unless otherwise specified.

### 2.2. Cell Lines and Culture Conditions

The human ccRCC cell lines, Caki-1 (ATCC^®^ HTB-46^™^) and Caki-2 (ATCC^®^ HTB-47^™^) were obtained from American Type Culture Collection (ATCC, Manassas, VA, USA). Cells were maintained in McCoy’s 5A (modified) medium supplemented with 10% FBS, 1% l-glutamine and 1% penicillin/streptomycin. Cells were cultured in a 37 °C humidified atmosphere containing 5% CO_2_ and 95% air. All methods were conducted in accordance with the relevant guidelines and regulations of the institutional biosafety committee.

### 2.3. Cell Viability Assay 

Cells were seeded at a density of 2 × 10^5^ cells per well in 6-well plates and incubated in complete medium. Next day, cells were either left untreated (control) or incubated with various concentrations of metformin (1, 2, 5, 10, 20, and 50 mM) for a further period of 48 h. A cell proliferation assay was then performed using Alamar Blue™ reagent according to the manufacturer’s recommendations. Alamar Blue™ stock solution was added at a ratio of 1:50 in the culture medium, and then cells were incubated for 4 h protected from direct light. Fluorescence was then read with 544 nm excitation and 590 nm emission wavelengths using a Synergy H1 microplate reader (BioTek Instruments, Winooski, VT, USA). Cell viability was assessed by fluorescence and values were normalized to control and expressed as the percentage of control as mean ± standard error of the mean (SEM).

### 2.4. Cell Cycle Analysis

Cells were seeded at the density of 2 × 10^5^ per well in 6-well plates. The next day, cells were either left untreated or incubated with metformin (concentrations ranging from 1 to 50 mM) for a period of 48 h. Cell cycle progression analysis was then performed using the Tali ^TM^ cell cycle assay protocol following the manufacturer guidelines. Briefly, cells were trypsinized, centrifuged and resuspended in PBS. Suspended cells were then fixed with 70% ice-cold ethanol and placed at −20 °C overnight. The following day, cells were stained with 200 µL of Tali ^TM^ cell cycle solution and incubated at room temperature for 30 min in the dark before being analyzed using a Tali^TM^ image-based cytometer (Life Technologies, Paisley, UK). 

### 2.5. Detection of Apoptosis

The annexin V-FITC apoptosis detection kit was used to assess apoptosis in both Caki-1 and Caki-2 cell lines. Briefly, cells were seeded at the density of 2 × 10^5^ cells/well in 6-well plates. The following day, cells were treated with various concentrations of metformin (2, 5, 10, 20, and 50 mM) for a further period of 48 h. Then, the supernatant was collected, and cells were trypsinized and suspended in 1× annexin V binding buffer at a concentration of 1 × 10^6^ cells/mL. Following this, 5 μL of annexin V and 2 μL of PI were added to the suspension of cells (100 μL containing 1 × 10^5^ cells). The suspension was then vortexed gently and incubated for 30 min at room temperature in the dark before being analyzed by flow cytometry using a BD LSR Fortessa cell analyzer^TM^ system (BD Biosciences, San Jose, CA, USA).

### 2.6. In Vitro Scratch Migration Assay 

The in vitro scratch migration assay was performed to quantify the rate of cell migration and was performed as described by Hulkower and Herber [30]. Briefly, cells were seeded in a 6-well culture plate at the density of 2 × 10^5^ cells/well and kept in the CO_2_ incubator. The next day, using a sterile 200 µL pipette tip, a thin scratch was made through the confluent monolayer of cells, and carefully the old culture medium and cell debris were aspirated. Cells were replenished with fresh medium. Finally, images were captured using optika vision image analyzer (Optika Srl, Via Rigla, Ponteranica, Italy) before and after metformin treatment (5, 10, 20, and 50 mM) at the following time points: 0, 6, and 24 h. The values were expressed as the percentage (%) of gap reduction compared to baseline (at 0 h time point).

### 2.7. Assessment of Autophagy

The Cyto-ID^®^ autophagy detection kit was used to monitor autophagy in both Caki-1 and Caki-2 cell lines. Briefly, cells were seeded at the density of 2 × 10^5^ cells/well in 6-well plates. The next day, cells were left untreated or incubated with various concentrations of metformin (10, 20 and 50 mM). After 48 h of drug treatment, cells were washed with PBS and incubated with a master-mix solution containing Cyto-ID^®^ green autophagy detection dye (1:1000), Hoechst 33342 (1 μM) and 5% FBS for 30 minutes at 37 °C. Then, cells were washed and immediately analyzed by flow cytometry using a BD LSR Fortessa cell analyzer^TM^ system. 

### 2.8. Western Blot Analysis

Western blotting was performed as described previously [31] to assess the effects of metformin on the expression of critical proteins involved in various signaling pathways such as AMPK, PTEN, AKT/mTOR, HIF-1α pathway, autophagy (light chain 3 (LC3)), EMT marker α-smooth muscle actin (α -SMA) and Wnt/β-catenin signaling pathway. Briefly, after 48 h of metformin treatment, the whole-cell lysate was collected in sodium dodecyl sulfate (SDS) sample lysis buffer (0.5 M Tris pH 6.8, 20% SDS and a cocktail of protease and phosphatase inhibitors) followed by sonication. Protein concentrations were determined in samples using the bicinchoninic acid assay (BCA). An equal amount of proteins (30 μg) were then loaded and separated on sodium dodecyl sulfate–polyacrylamide (SDS–PAGE) gels and later transferred to a polyvinylidene difluoride (PVDF) membrane. After blocking, blots were incubated overnight with respective primary antibodies (Cell Signaling Technology), washed three times with a mixture of Tris-buffered saline (TBS) and polysorbate 20 (T-TBS), and then incubated with appropriate horseradish peroxidase-conjugated secondary antibodies (Cell Signaling Technology) for 1 h. Enhanced chemiluminescence (ECL) reagent (Abcam) was used to visualize the proteins bands using FlourChem^TM^ M system (ProteinSimple, San Jose, CA, USA). The AlphaView software (Protein Simple, San Jose, CA, USA) was used to quantify protein band intensities. 

### 2.9. Statistical Analysis

The results were expressed as the mean ± SEM. One-way or two-way analysis of variance (ANOVA) tests, as appropriate, followed by Tukey’s or Bonferroni multiple comparisons post-hoc tests were performed using Prism 7 (GraphPad, San Diego, CA, USA) to compare the groups. *P* < 0.05 was considered to be statistically significant. 

## 3. Results

### 3.1. Metformin Causes a Reduction in Cell Viability in both Caki-1 and Caki-2 Cell Lines

**Figure 1:**
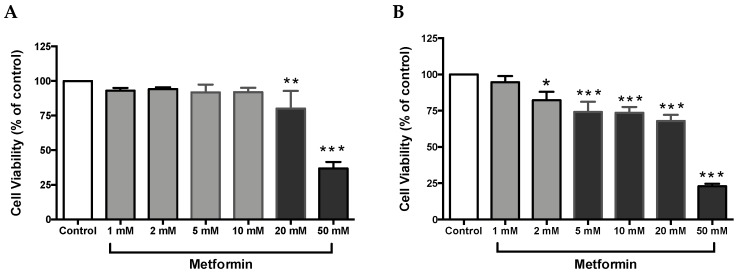
Metformin reduced cell viability in Caki-1 and Caki-2 renal cancer cell lines. (**A**) Caki-1 and (**B**) Caki-2 cells were incubated in the absence (control) or presence of metformin (1, 2, 5, 10, 20, and 50 mM) for 48 h. Cell viability was analyzed using Alamar Blue™ assay, and the fluorescence values were normalized to control and expressed as the percentage of control. Data are presented as mean ± SEM (*n* = 4–5 per group). * *P* < 0.05; ** *P* < 0.001; and *** *P* < 0.0001 vs. control.

Cell proliferation is an integral part of tumor promotion and progression, and it manifests with the altered expression of various proteins [32]. Thus, to investigate whether metformin could inhibit cell growth in ccRCC, both Caki-1 and Caki-2 cell lines were incubated in the absence and presence of metformin (1–50 mM) for a period of 48 h. As shown in Figure 1A, no major changes in cell viability were observed in metformin-treated Caki-1 cells up to the concentration of 10 mM (92 % of cells were still viable); however, a significant dose-dependent decrease in cell viability was observed with higher concentrations (20 mM and 50 mM (84% and 42% respectively)). Interestingly, metformin-treated Caki-2 cells showed a significant and dose-dependent decrease in cell viability at all concentrations except the lowest (i.e., 82% in 2 mM, 74% in 5 mM, 73% in 10 mM, 68% in 20 mM, and 23% in 50 mM) as shown in Figure 1B. Altogether, our findings indicated a differential sensitivity of Caki-1 and Caki-2 cells to cell viability reduction caused by metformin, with Caki-2 being more sensitive to lower doses of metformin. 

### 3.2. Metformin Causes G0/G1 Phase Cell Cycle Arrest in Caki Cell Lines

**Figure 2:**
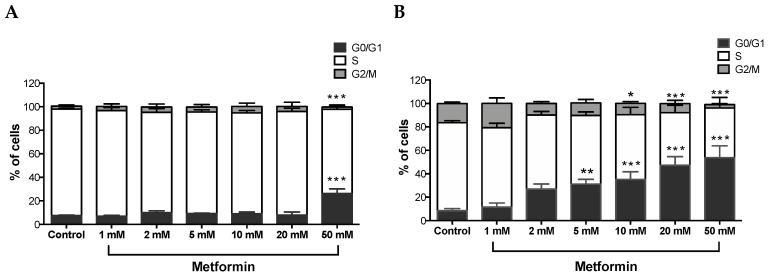
Metformin-induced G0/G1 cell cycle arrest in Caki-1 and Caki-2 cells. Cell cycle progression was analyzed in (**A**) Caki-1 and (**B**) Caki-2. Cells were incubated in the absence (control) or presence of metformin (1, 2, 5, 10, 20, and 50 mM) for 48 h. Histograms represent the percentage of cells measured at each of the different phases of the cell cycle in each treatment group. Data are presented as mean ± SEM and were analyzed by two-way ANOVA, followed with Tukey’s multiple comparison tests (*n* = 4–6 per group). * *P* < 0.05; ** *P* < 0.001; and *** *P* < 0.0001 vs. control.

A Tali ^TM^ cell cycle assay kit was used to determine the percentage of cells in each cell cycle phase following exposure of ccRCC cells to varying concentrations of metformin for 48 h. As shown in Figure 2A, no changes were observed with Caki-1 cells up to 20 mM of metformin with only 12% in 2 mM, 14% in 5 mM, 15% in 10 mM, and 18% in 20 mM of cells displayed cell cycle arrest at G0/G1; however, at the 50 mM dose, around 34% of cells showed substantial cell cycle arrest at G0/G1 compared to the control (7%). In contrast, Caki-2 cells demonstrated strong dose-dependent (5–50 mM) cell cycle arrest at G0/G1 phase in the presence of metformin (i.e., 31% in 5 mM, 35% in 10 mM, 47% in 20 mM, and 54% in 50 mM compared to 8% in the control group; Figure 2B). These results further suggest that Caki-2 cells are more susceptible to the antiproliferative actions of metformin compared with Caki-1 cells.

### 3.3. Differential Apoptotic Response to Metformin Treatment in Caki-1 and Caki-2 Cells 

**Figure 3:**
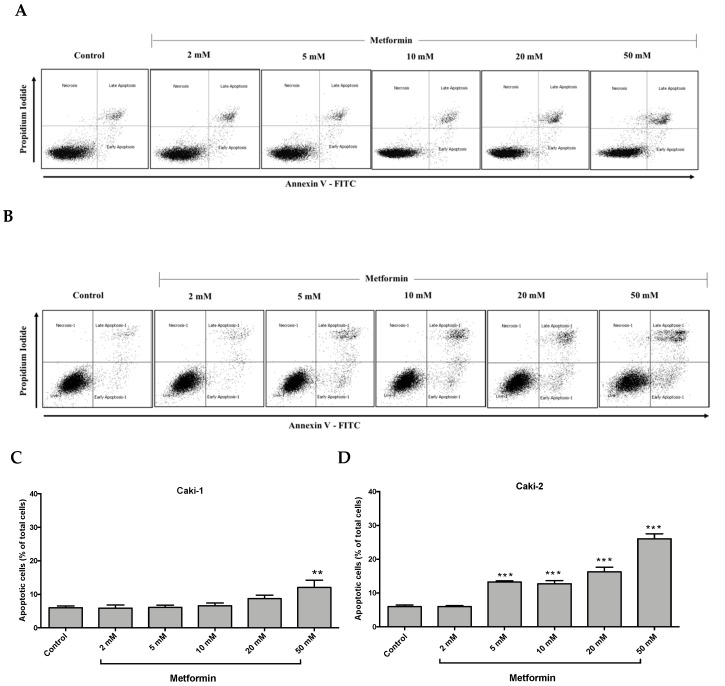
Metformin selectively induced a stronger apoptotic response in Caki-2 cells compared to Caki-1 cells. Flow cytometry analysis of apoptosis in (**A**,**C**) Caki-1 and (**B**,**D**) Caki-2. Cells were incubated in the absence (control) or presence of metformin (1, 2, 5, 10, 20, and 50 mM) for 48 h, and then apoptosis was determined by double labeling of cells with annexin V-FITC/propidium iodide staining, followed by flow cytometry analysis. (**C**,**D**) Histograms represent pooled data from four independent experiments per group. Data are presented as mean ± SEM. * *P* < 0.05; ** *P* < 0.001; and *** *P* < 0.0001 vs. control.

It is well-documented that oncogenic mutations can disrupt apoptosis and deregulate apoptotic signaling pathways, thereby enabling cancer cells to evade programmed cell death and subsequently leading to uncontrolled cell growth and migration [33]. We investigated the impact of metformin treatment on the induction of apoptosis in Caki-1 and Caki-2 cells. As depicted in Figure 3A,C, metformin failed to induce apoptosis in Caki-1 cells up to 20 mM, and only the highest concentration of metformin (i.e., 50 mM) caused a moderate induction of apoptosis (12%). However, a significant and dose-dependent induction of apoptosis was observed in Caki-2 cells starting from 5 mM up to 50 mM (26%) compared to the control (Figure 3B,D). These data further support that Caki-2 cells were more sensitive to the antineoplastic effects of metformin compared with Caki-1 cells. 

### 3.4. Metformin Suppresses Migration of Both Caki-1 and Caki-2 Cells 

**Figure 4:**
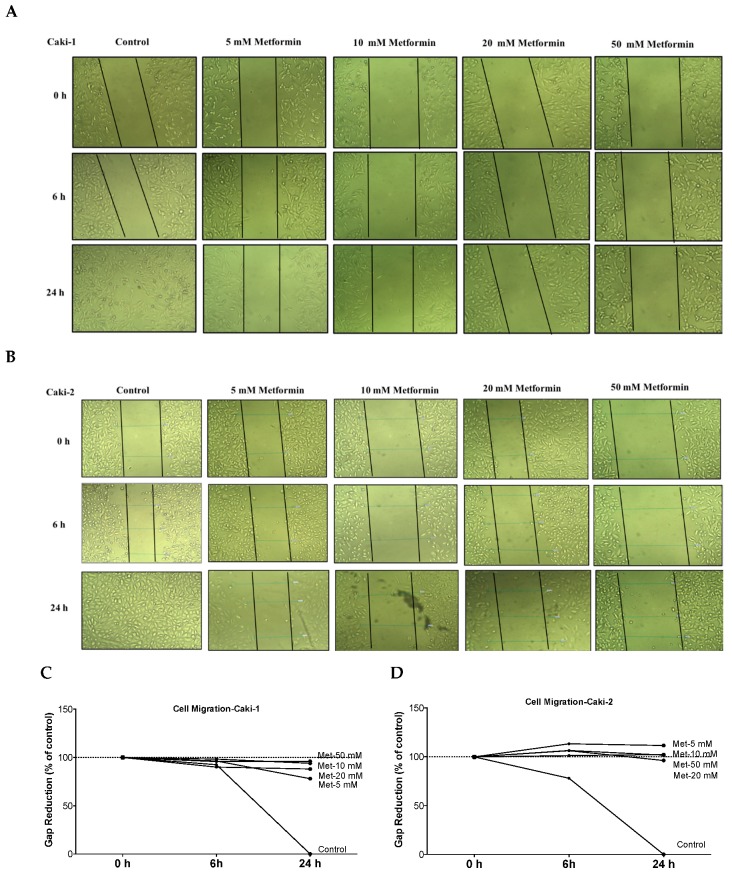

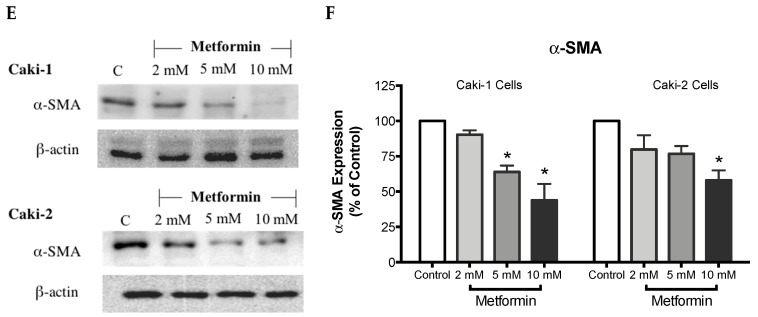
Metformin inhibited cell migration and invasion in both Caki-1 and Caki-2 cells. In vitro scratch migration assay at 0, 6 and 24 h following metformin treatment (5, 10, 20, and 50 mM) are shown in (**A**) Caki-1 cells (**B**) and Caki-2 cells. Images are representative of three independent experiments in each group. Pooled quantified normalized data expressed as gap reduction (percentage of control) is presented for (**C**) Caki-1 and (**D**) Caki-2. (**E**) Western blot analysis of the expression of α-SMA in Caki-1 and Caki-2. Images are representative of *n* = 4–5 per group. (**F**) Histograms represent pooled densitometry data normalized to β-actin expressed as the percentage of control. Data are presented as mean ± SEM. * *P* < 0.05 vs. control.

The ability of cancer cells to metastasize is principally determined by the cells’ capability to undergo changes and reorganize their morphology through processes, such as EMT, which confers cancer cells increased migratory capacity and invasiveness, and promotes tumor metastasis [34]. Moreover, studies in surgically-resected renal tissues from patients with ccRCC revealed the existence of cancer-associated fibroblasts, which can also promote tumor progression and metastasis through various mechanisms including induction of EMT [35,36]. Thus, we quantified the effect of metformin on cell migration rate using a scratch migration assay. We found that metformin treatment noticeably inhibited the capacity of wound healing in both Caki-1 (Figure 4A,C) and Caki-2 cells (Figure 4B,D), suggesting that metformin could effectively inhibit cell migration and invasion in both cell types whereas untreated Caki-1 and Caki-2 cells (controls) demonstrated a higher capacity to migrate and invade (gap closed within 24 h). These data indicate that metformin treatment equally inhibited cancer cell migration in both Caki-1 and Caki-2 cells.

α-SMA is a highly conserved protein involved in cell motility, structure and integrity. Moreover, α-SMA is commonly used as a marker of myofibroblast differentiation, and it plays a substantial role in cancer progression. Thus, we assessed the protein expression of α-SMA to decipher the antimigratory effect of metformin in ccRCC. Interestingly, as depicted in Figure 4E,F, metformin caused dose-dependent repression of α-SMA’s protein expression in both Caki-1 and Caki-2 cells. Intriguingly, Caki-1 cells exhibited a more pronounced decrease in α-SMA’s protein expression, which was observed with doses as low as 5 and 10 mM; whereas, in Caki-2 cells, the reduction of α-SMA’s expression was observed with higher doses (10 mM) as shown in Figure 4F. From these findings, it is evident that metformin was effective in blunting cell migration and reversing EMT in both Caki-1 and Caki-2 cells.

### 3.5. Metformin Represses Hypoxia-Inducible Factor-1α Protein

**Figure 5:**
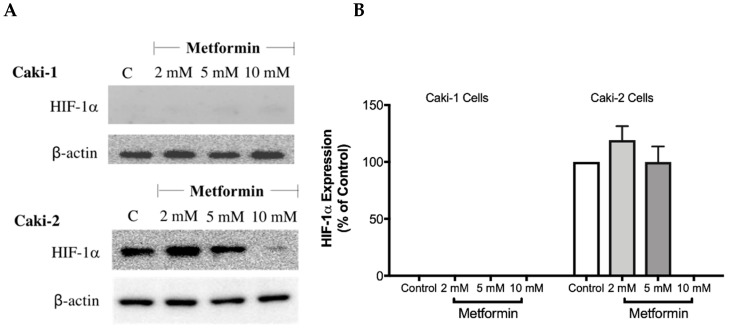
Metformin reduced the expression of HIF-1α in Caki-2 renal cancer cells. (**A**) Shows a Western blot analysis of HIF-1α in Caki-1 and Caki-2 cells incubated in the absence (control) or presence of metformin (2, 5, and 10 mM). Images are representative of three independent experiments per group. (**B**) Histograms represent pooled densitometry data normalized to β-actin expressed as the percentage of control. Data are presented as mean ± SEM.

The overexpression of HIF-1α protein has been shown to be involved in the development and progression of ccRCC due to mutations in the *VHL* gene [28]. Therefore, targeting HIF-1α protein might be a promising approach. To investigate whether metformin could affect the expression of HIF-1α, Western blot analysis was performed in both cell lines in the absence and presence of metformin (2, 5, and 10 mM). Intriguingly, as shown in Figure 5A,B, there was no expression of HIF-1α protein with Caki-1 cells; however, Caki-2 cells expressed HIF-1α protein. Importantly, in the presence of metformin (10 mM), the protein expression of HIF-1α was reduced in Caki-2 cells compared to the control (Figure 5A). In addition, we also evaluated the expression of HIF-2α protein (data not shown); however, we did not observe any expression of this protein in both cell lines. These results showed that Caki-2 cells, which are known to express wild-type pVHL, expressed high levels of HIF-1α protein as expected; however, Caki-1 cells although classified as metastatic ccRCC and reported to be harboring wild-type *VHL*, did not express HIF-1α protein in our study. 

### 3.6. Metformin Treatment Causes AMPK Activation and Inhibition of Akt/mTOR Axis in ccRCC Cell Lines

It is well-documented that AMPK is a cellular metabolic protein that has been connected to the PI3K/Akt/mTOR signaling axis, and the latter is involved in various cellular signaling pathways that are vital for cell growth and differentiation [37,38]. As Metformin is a known AMPK activator, we investigated the effect of metformin treatment on AMPK and the Akt/mTOR signaling pathway in Caki cell lines. 

**Figure 6:**
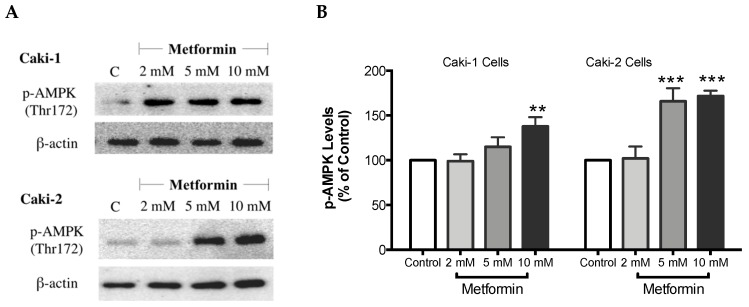

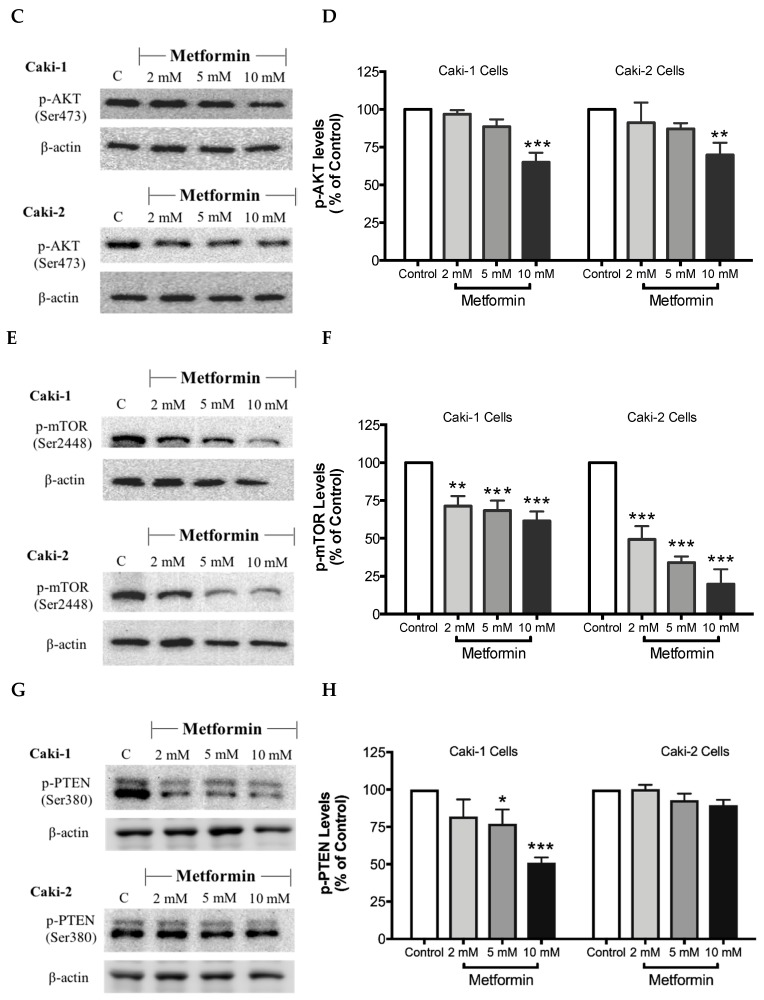
Metformin activated AMPK and reduced the activation of the Akt/mTOR axis in both Caki-1 and Caki-2 renal cancer cells. Western blot analyses are shown for (**A**,**B**) p-AMPK (Thr172), (**C**,**D**) p-Akt (Ser473), (**E**,**F**) p-mTOR (Ser2448), and (**G**,**H**) PTEN (Ser380) in Caki-1 and Caki-2 cells incubated in the absence (control) or presence of metformin (2, 5, and 10 mM). Images are representative of three independent experiments per group. (**B**,**D**,**F**,**H**) Histograms represent pooled densitometry data normalized to β-actin expressed as the percentage of control. Data are presented as mean ± SEM. * *P* < 0.05; ** *P* < 0.001; and *** *P* < 0.0001 vs. control.

As expected, treatment with metformin induced a dose-dependent activation of AMPK in both Caki-1 and Caki-2 cells in comparison to the control, as shown in Figure 6A,B. Significant activation of AMPK was observed with metformin at 5 mM (166%) and 10 mM (172%) in Caki-2; however, Caki-1 cells exhibited a significant activation of AMPK only with the highest dose of metformin (10 mM (138%)), as shown in Figure 6B. On the other hand, as depicted in Figure 6C,D, our results also revealed a dose-dependent reduction of Akt phosphorylation in both cell lines in the presence of metformin, with the most prominent effect observed at the concentration of 10 mM of metformin. Furthermore, significant repression of mTOR phosphorylation was observed in a dose-dependent manner in both cell lines; however, Caki-2 cells were more sensitive in comparison with Caki-1 cells to the effect of metformin as shown in Figure 6E,F. These findings suggest that the antineoplastic effect of metformin is associated with the activation of AMPK, which negatively regulates mTOR, a key mediator in the PI3K/Akt signaling pathway. In addition, we investigated if the effect of metformin was dependent or independent of PTEN protein, which is a natural inhibitor of the PI3K/Akt pathway [39]. Our results revealed that the metformin-induced suppression of PTEN phosphorylation was more prominent in Caki-1 cells compared to Caki-2 cells as shown in Figure 6G,H.

### 3.7. Metformin Reduces the Expression of Autophagy-Related Protein LC3

**Figure 7:**
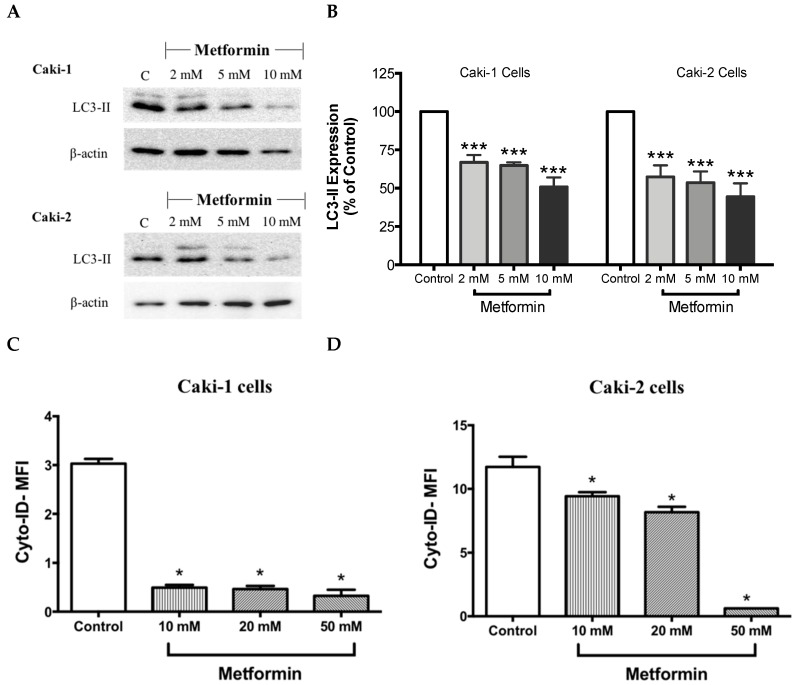
Metformin blunted autophagy induction in both Caki-1 and Caki-2 renal cancer cells. (**A**) Western blot analysis of LC3-II in Caki-1 and Caki-2 cells incubated in the absence (control) or presence of metformin (2, 5 and 10 mM). Images are representative of three independent experiments per group. (**B**) Histograms represent pooled densitometry data normalized to β-actin expressed as the percentage of control. Data are presented as mean ± SEM. Results of autophagy assessment by flow cytometry analysis using a Cyto-ID^®^ are shown for (**C**) Caki-1 and (**D**) Caki-2 cells. (**C**,**D**) Histograms represent pooled data from three independent experiments per group. Data are presented as mean ± SEM. * *P* < 0.05; ** *P* < 0.001; and *** *P* < 0.0001 vs. control.

It is well-known that at the molecular level, autophagy regulates various cell death and survival signaling pathways that can, in turn, decide the fate of tumor cells [16,40]. Moreover, studies have shown that mTOR negatively regulates autophagy in cancer cells [40]. Hence, one could expect an induction of autophagy process in the presence of metformin; however, surprisingly in our study, metformin failed to induce the expression LC3-II, a key marker of autophagy, in both Caki-1 and Caki-2 cells (Figure 7A,B). Instead, a paradoxical response with a low level of expression of LC3-II autophagic marker was observed in both cell lines, as shown in Figure 7A,B. Autophagy inhibition was further confirmed by flow cytometry analysis using a Cyto-ID^®^ kit where a significant inhibition of autophagic flux was observed in both Caki-1 and Caki-2 cells in the presence of metformin compared to controls (Figure 7C,D).

### 3.8. Metformin Decreases the Expression of β-Catenin, an Important Regulator of the Canonical Wnt Signaling Pathway

**Figure 8:**
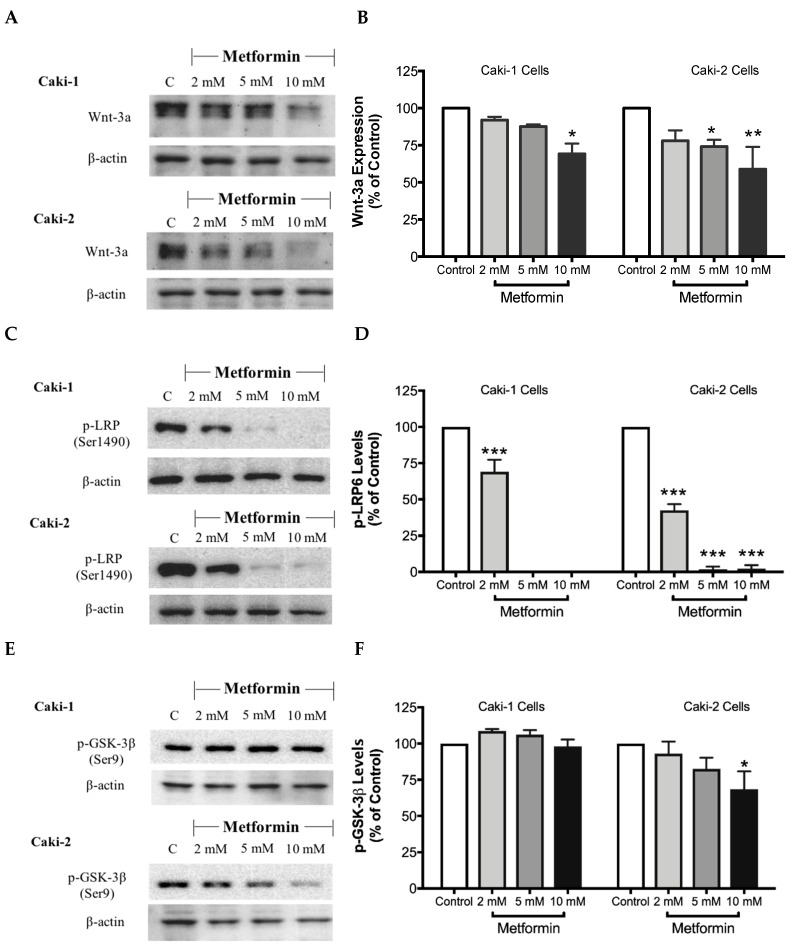

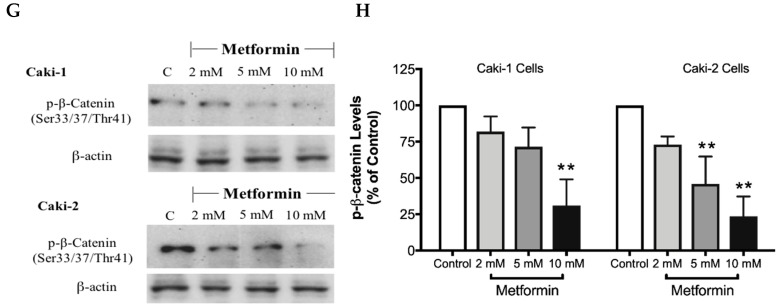
Metformin inhibited Wnt/β-catenin signaling in both Caki-1 and Caki-2 renal cancer cells. Western blot analyses are shown for (**A**,**B**) Wnt3a, (**C**,**D**) p-LRP6 (Ser1490), (**E**,**F**) GSK-3β (Ser9) and (**G**,**H**) p-β-catenin (Ser33/37/Thr41) protein in Caki-1 and Caki-2 cells incubated in the absence (control) or presence of metformin (2, 5, and 10 mM). Images are representative of three independent experiments per group. (**B**,**D**,**F**,**H**) Histograms represent pooled densitometry data normalized to β-actin expressed as the percentage of control. Data are presented as mean ± SEM. * *P* < 0.05; ** *P* < 0.001; and *** *P* < 0.0001 vs. control.

The canonical Wnt/β-catenin signaling pathway plays a critical role in various cellular and developmental processes, and its aberrant activation has been linked with some cancers including ccRCC [41]. Studies in ccRCC cell lines such as A-498, A704, and Caki-2 cells revealed a constitutive activation of the Wnt/β-catenin pathway with upregulation of β-catenin, which contributes to promoting excessive cell proliferation and differentiation [42]. Subsequently, targeting the Wnt/β-catenin signaling pathway might be a potential strategy to treat renal cancer. Thus, we evaluated here the impact of metformin treatment on the Wnt/β-catenin pathway. Our results showed a dose-dependent decrease in the protein levels of Wnt3a (Figure 8A,B), phospho-LRP6 (Figure 8C,D), and phospho-β-catenin (Figure 8G,H), in the presence of metformin in both Caki-1 and Caki-2 cells. However, the inhibition of this pathway was more pronounced in Caki-2 cells compared with Caki-1 cells. Together, these results further suggest that Caki-2 cells are more sensitive to metformin treatment compared to Caki-1 cells (Figure 8).

## 4. Discussion

In the current study, we investigated the potential anticancer activity of metformin against human ccRCC cell lines, Caki-1 and Caki-2. In addition, we examined the possible signal transduction mechanisms through which metformin could exert its antineoplastic effects against ccRCC. There is an increasing body of evidence that metformin can inhibit cell proliferation and differentiation in various types of cancers such as cancers of the lung, prostate, liver, and pancreas [43,44,45,46]. Consistent with these previous studies, our findings demonstrated that metformin was capable of blunting cell growth in both Caki-1 and Caki-2 cells. However, a significant and dose-dependent response to metformin treatment was observed with Caki-2 cells in comparison with Caki-1 cells, with Caki-2 being more sensitive, indicating a difference in sensitivity between the two cell lines toward the action of metformin. Consistent with our observations, Kalogirou et al. [29] demonstrated that Caki-1 cells were less sensitive towards metformin treatment. Furthermore, Zhang et al. [47] reported that metformin decreased the cell viability of Caki-2 and 786-O cells in a dose-dependent manner. Additionally, Zhong et al. [48] also observed that metformin inhibited the proliferation of 786-O renal cancer cells.

Cell cycle analysis showed that metformin treatment caused G0/G1 cell cycle arrest in both Caki-1 and Caki-2 cells compared to controls. However, our data showed a strong and significant dose-dependent G0/G1 phase arrest in Caki-2 cells in comparison with Caki-1 cells. Consistent with our findings, Liu et al. [3] demonstrated that metformin induced G0/G1 cell cycle arrest in Caki-2 and 786-O cells. Similarly, Xie et al. [49] reported that metformin produced G0/G1 phase arrest in human renal cell carcinoma cell line ACHN. To correlate cell cycle results and apoptosis, we assessed the apoptotic effect of metformin because in some cases cell cycle arrest may not necessarily be followed by cell death or apoptosis. Our findings showed that metformin failed to induce cytotoxicity in Caki-1 cells, while a significant cytotoxic effect was observed with Caki-2 cells, indicating a differential sensitivity of the two cell lines towards metformin. A study by Zhong et al. [48] revealed that metformin induces cell death in 786-O renal cancer cells under conditions of low nutritional status, which taken together with our results indicate a differential sensitivity of ccRCC cells to metformin, adding to the heterogeneity of this cancer.

It is well-known that cell migration and invasion play a crucial role in the metastasis of cancer cells. Our results from the scratch migration study indicated that metformin inhibited cell migration of both ccRCC cell lines used. Furthermore, the Western blot analysis results revealed dose-dependent repression of α-SMA protein expression, further supporting the antimigratory effect of metformin in ccRCC cell lines (Caki-1 and Caki-2). 

To explore the underlying molecular mechanism(s) involved in the antineoplastic effects of metformin in Caki-1 and Caki-2 cell lines, we investigated the impact of metformin treatment on various crucial signaling pathways involved in the progression of ccRCC: HIF-1α, AMPK, Akt/mTOR and Wnt/β-catenin pathways. It is well-documented that the mutation of the *VHL* tumor suppressor gene promotes the constitutive activation of HIF-1α and HIF-2α subunits in RCC [17]. Intriguingly, our study demonstrated two critical differences between Caki-1 and Caki-2 cells. Firstly, although there are close similarities between the two isoforms of HIF-α, only HIF-1α was constitutively expressed in Caki-1 and Caki-2 cells. Secondly, our results showed that the expression of HIF-1α protein was predominantly higher in Caki-2 cells compared to Caki-1 cells and that metformin treatment (10 mM) caused significant repression of its expression compared to the control. Consistent with our findings, Raval et al. [17] observed contrasting properties of HIF-1α and HIF-2α on the growth of 786-O cells in tumor xenografts. 

There has been increasing evidence interconnecting the significance of cancer and metabolism. Cancer cells need to continually synthesize new proteins to support their rapid rate of proliferation and differentiation, ultimately causing an imbalance in the cellular energy levels, which can be detected by AMPK [50,51]. Therefore, targeting AMPK might be an effective approach to treat cancer, either alone or in combination. In the present study, as expected, metformin upregulated the phosphorylation of AMPK in both Caki-1 and Caki-2 cells. However, Caki-2 cells expressed higher levels of phosphorylated AMPK compared to Caki-1 cells. AMPK has been linked to other essential pathways implicated in cell growth like the PI3K/Akt/mTOR axis, which was found to be activated in ccRCC [52]. Notably, by activating AMPK, metformin decreased the phosphorylation and subsequently the activation of both Akt and mTOR in both Caki-1 and Caki-2 cells. Interestingly, metformin reduced the phosphorylation of mTOR more strongly in Caki-2 cells compared to Caki-1, which correlates with a higher level of phosphorylated AMPK observed with Caki-2 cells. Liu et al. [3] confirmed in a study that metformin induced the activation of AMPK and inhibited mTOR activation in 786-O and OS-RC-2 ccRCC cell lines. In addition, Liu et al. [3] has shown, in a murine xenograft model, that daily treatment of mice with metformin prevented ccRCC tumor growth. Although many previous studies have demonstrated the significance of mTOR as an essential negative regulator of autophagy induction [53], to our surprise, our results were contrasting in both Caki-1 and Caki-2 cells as metformin caused a significant inhibition of autophagy (measured using LC3-II levels) compared to a control. It is reasonable to speculate that metformin may have caused this paradoxical effect on autophagy through an AMPK-independent mechanism. Depending on the dose and duration of treatment, metformin was found to act via both AMPK-dependent and AMPK-independent mechanisms [54]. Inhibition of autophagy has been reported in other studies to also contribute to cancer cell death in ccRCC [55]. For instance, Singla et al. [56] explored the effect of combining temsirolimus (mTOR inhibitor) and chloroquine (autophagy inhibitor) to ameliorate anticancer activity against ccRCC in EMT-transformed metastatic cells. Moreover, emergent evidence indicates that in a nutrient-limited microenvironment, autophagy can promote tumor growth [57,58]. Thus, inhibition of autophagy is regarded as an effective strategy to suppress tumor progression in certain cancers. Taken together with our findings, inhibition of autophagy might represent a potential mechanism by which metformin elicits its antineoplastic effects against ccRCC.

Besides *VHL* mutation with subsequent overactivation of HIF-1α and mTOR signaling pathways, other signaling pathways may also play a vital role in the development and progression of ccRCC. Of particular interest, Wnt/β-catenin pathway and its dysregulation with subsequent overactivation of β-catenin have been implicated in ccRCC pathogenesis [42,59]. Therefore, the inhibition of Wnt/β-catenin signaling pathway might emerge as a novel alternative therapeutic target to suppress ccRCC progression. Interestingly, our current study revealed that the metformin-treated cells exhibited a lower level of expression of major modulators of the Wnt/β-catenin pathway including β-catenin in both Caki-1 and Caki-2 cell lines compared to controls, which may, in turn, contribute to the antineoplastic effects of metformin in ccRCC.

## 5. Conclusions

Our findings demonstrate that metformin may be a potential drug of choice in the treatment of ccRCC. Differential responses were observed between the two cell lines studied, with Caki-2 cells being more sensitive to metformin compared with Caki-1 cells, indicating the complexity of this cancer. The discrepancy in sensitivity to metformin between the two cell types could be linked to the differential expression of HIF-1α despite that both cell lines were reported to carry a wild-type *VHL*. Furthermore, we highlighted here that antineoplastic effects of metformin against ccRCC are, at least partly, based on its ability to activate AMPK and the subsequent inhibition of Akt/mTOR axis in addition to a focal effect on the inhibition of the Wnt/β-catenin pathway. Nevertheless, our data do not rule out other AMPK-independent mechanisms. Because of the heterogeneity of ccRCC and the molecular differences between its subtypes, additional studies are warranted to further harness the potential use of metformin as an antitumor agent in ccRCC.
